# Spatial Frequency Information Modulates Response Inhibition and Decision-Making Processes

**DOI:** 10.1371/journal.pone.0076467

**Published:** 2013-10-21

**Authors:** Sara Jahfari, K. Richard Ridderinkhof, H. Steven Scholte

**Affiliations:** 1 Department of Psychology, University of Amsterdam, Amsterdam, The Netherlands; 2 Cognitive Science Center Amsterdam, University of Amsterdam, Amsterdam, The Netherlands; McGill University, Canada

## Abstract

We interact with the world through the assessment of available, but sometimes imperfect, sensory information. However, little is known about how variance in the quality of sensory information affects the regulation of controlled actions. In a series of three experiments, comprising a total of seven behavioral studies, we examined how different types of spatial frequency information affect underlying processes of response inhibition and selection. Participants underwent a stop-signal task, a two choice speed/accuracy balance experiment, and a variant of both these tasks where prior information was given about the nature of stimuli. In all experiments, stimuli were either intact, or contained only high-, or low- spatial frequencies. Overall, drift diffusion model analysis showed a decreased rate of information processing when spatial frequencies were removed, whereas the criterion for information accumulation was lowered. When spatial frequency information was intact, the cost of response inhibition increased (longer SSRT), while a correct response was produced faster (shorter reaction times) and with more certainty (decreased errors). When we manipulated the motivation to respond with a deadline (i.e., be fast or accurate), removal of spatial frequency information slowed response times only when instructions emphasized accuracy. However, the slowing of response times did not improve error rates, when compared to fast instruction trials. These behavioral studies suggest that the removal of spatial frequency information differentially affects the speed of response initiation, inhibition, and the efficiency to balance fast or accurate responses. More generally, the present results indicate a task-independent influence of basic sensory information on strategic adjustments in action control.

## Introduction

More often than not, we use the available sensory information from our environment to select or control our actions. Perceptual decision-making is vastly becoming a central theme in psychology and neurosciences [1]–[4]. The process of perceptual decision-making entails the voluntary selection of a motor response from a set of response alternatives, based on information gathered from the sensory system. Influential work in this field has reported perceptual judgment alternations through the manipulation of stimulus difficulty or coherence [5], [6], the reliability of visual evidence [7], and the integration of sensory evidence towards decision outcomes [8], [9]. In the field of action control, the perceptual overlap between stimuli that trigger response initiation or inhibition affected both performance time and the monitoring of response conflict [10]. In addition, the efficiency to control ones actions was related to the complexity of perceptual information [11], and lateralization differences with the presentation of verbal stimuli [12]. Together, these findings highlight the importance of perceptual information during action formation and control. However, still much can be learned about how perceptual information affects psychological processes that underlie the initiation or regulation of controlled behavior. The present study set out to examine how variance in the quality of perceptual information affects the suppression and initiation of planned actions.

Traditionally, experimental psychology has used paradigms that tap into our ability to suppress a planned response, or to respond strategically with an a priori motivated plan (e.g., fast or accurate), to study mechanisms of response inhibition or decision-making. While previous work has shown relationships between perceptual processing and motor control (e.g., [9]), intuitively, the encoding of visual information (e.g., image shape or details) and action control (e.g., strategic responding, or the suppression of planned responses) appear to be two separate psychological processes. This relationship becomes apparent when considering an example where one is driving a car without wearing glasses, and has less detailed information about his or her surroundings. Here, to prevent accidents, one drives more slowly (carefully acquiring global information by increasing reaction time), and is more ready or prepared to stop for unexpected changes (more efficient response inhibition). Consistently, work on the relationship between perception time an visual information has reported the processing of high spatial frequency information (containing mainly detailed scene features) to demand more time, when compared to the processing of low spatial frequency information (containing more global scene features) [13]. In addition, subjects are capable of categorizing global image properties with less viewing time than basic-level categorization [14]. Moreover, research has shown the fast processing of global scene information to interfere with response selection [15], and to increase response conflict [16]. These effects were not observed for more slowly processed detailed or local features. Together, these examples suggest that global or local attributes of a perceptual scene should differentially affect both perception time and higher order cognitive processes such as response selection and inhibition.

One prominent field where qualitative differences in perceptual information have been linked to changes in perceptual judgements is face recognition [17]–[19]. Research within this field has suggested that, among others, the categorization and recognition of faces is made possible through the physical characteristics of available spatial frequency information [20]–[23]. Importantly, fast global shape processing has been linked to low-spatial-frequency information processed via the magnocellular pathways, whereas high-spatial-frequency information has been related to a slower processing of fine details via the slow parvocellular pathways [24], [25]. The magnocellular and parvocellular pathways form two distinct streams of processing that mediate the analysis of different types of visual information. The receptive fields of neurons in the magnocellular pathways are large and respond to low spatial frequencies. This stream projects predominantly into parietal cortex and is important for spatial and motion processing. In contrast, the receptive fields of neurons in the parvocellular pathway are smaller and respond to higher spatial frequencies. This stream projects into temporal cortex and is important for object recognition and categorization. Although, spatial frequency information is known to affect response selection, or stimulus identification/categorization, effects of spatial frequency information on higher order processes like decision-making or inhibitory control remain largely unknown.

In this study, we specify how spatial frequency information modulates the decision process i) to *withhold* a planned response, *and* ii) to *select* a response. To examine how spatial frequency affects underlying processes of response inhibition, we used a modified version of the stop-signal task where incidental stop-signals designate that a planned response has to be withdrawn[26]–[33]. Here, we replaced the traditional left- or rightward arrows (for the go task) with male or female faces containing only low-, only high-, or all spatial frequencies. Theoretically, action regulation within the stop-task has been described as an independent race between the go (response initiation) and the stop (response inhibition) response [34], [35]. Therefore, within this framework, noisy or degraded sensory information might have a differential effect on the strategic decision to inhibit or initiate planned actions. To examine how spatial frequency information affects strategic decision making, we used a speed-accuracy experiment where participants identified the gender of the presented faces, with an emphasis on either speed or accuracy [36]–[38]. While speed-accuracy paradigms have been used previously to study relationships between sensory evidence and response regulation, no work has described how different types of spatial frequency information (processed at different speeds) affect latent processes that underly strategic action selection. In addition, work in the field of perceptual decision-making has predominantly focused on processes of response regulation and planning, and not on response inhibition, where a planned response (based on certain or uncertain sensory information) has to be withdrawn.

**Figure 1:**
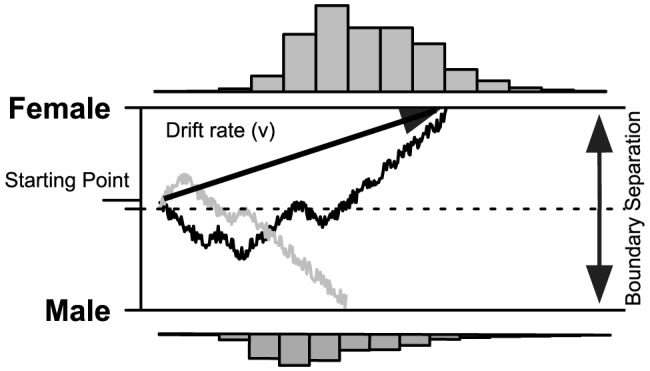
Graphical outline of the Drift Diffusion Model (DDM). The two sample paths represent accumulation of evidence from a presented female face stimulus; resulting in either a correct (black line) or incorrect response (grey line). As shown in the RT histograms, responses more often accumulate towards a correct choice (above) than an incorrect choice (below). Drift rate (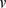) represents the average amount of evidence accumulated per time unit. Boundary separation (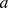) represents how much evidence is needed before a definitive response is made.

To establish how spatial frequency information affects latent processes that underly response selection, or inhibition, we used a hierarchical version of the drift-diffusion model for decision-making [39]–[42]. This formal cognitive process model (Figure 1), allows for the decomposition of correct and incorrect RT data into separate decision and non-decision components [43]–[45]. Therefore, this approach allowed us to establish how the degradation of visual information affects the ease of evidence accumulation (“drift”), and/or strategic adjustments in the amount of evidence required before a decision threshold is reached (“boundary”). In all experiments, performance differences due to stimulus manipulations were expected to affect both the speed of information accumulation (“drift”), and response cautiousness (“boundary”). Due to the slower processing of finer details, the removal of especially low spatial frequency information (in HSF faces) was expected to have the largest effect on the speed of information accumulation (“drift”). The slower the progression of evidence accumulation, the greater the cost of acquiring additional information from sensory regions [46]. We therefore expected the selective removal of spatial frequencies to affect both drift rate and response “boundary” for evidence accumulation. That is, when the speed of information accumulation becomes very low - due to the removal of spatial frequency information - we expect participants to lower the required accuracy levels to afford timely action selection.

**Figure 2:**
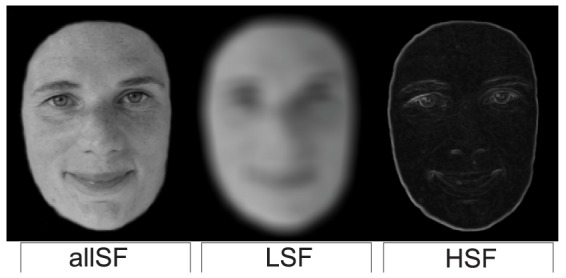
Example of a face with all spatial frequency information (allSF), only low spatial frequency information (LSF), or only high spatial frequency information (HSF). Note that the prints underestimate the contrasts used in the current experiment, especially for the LSF and HSF pictures.

In all experiments, we manipulated the frequency information in faces used for perceptual judgments during inhibitory control and strategic decision-making (Figure 2). Specifically, three sets of faces containing either high (HSF), low (LSF), or both spatial frequencies (allSF) were used as stimuli in a modified version of the stop- and speed-accuracy task. The first experiment focused on response inhibition, and examined how different types of spatial frequency information affect the efficiency to suppress a planned response. In the stop-signal task, the efficiency of inhibitory control is indicated by the time needed to withhold a planned response (stop signal reaction time; SSRT). Given the car example, reduced levels of spatial frequency were expected to increase reaction times during go trials, while, at the same time, improving the efficacy to stop (lower SSRT) when an unexpected stop-signal is sounded, improving the efficacy to stop (lower SSRT) when an unexpected stop-signal is sounded, analogous to effects of spatial attention reported by [47]. Experiment 2 was designed to replicate findings from the first experiment with the stop-signal task, and extend these to the field of strategic decision-making. Using a modified speed-accuracy task with the altered faces, degraded levels of information were especially expected to affect response strategies when participants were motivated to be accurate (e.g., to prevent an accident). A third experiment was conducted to assess the reliability of effects found during strategic decision-making.

## Results

### Experiment 1

**Figure 3:**
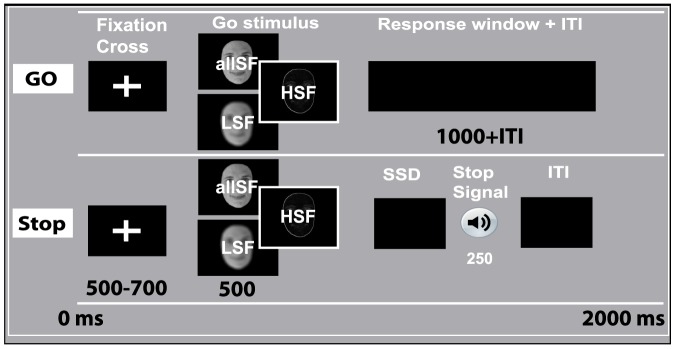
Visual stop-task. Each trial started with a white fixation-cross followed by a male or female face stimulus, indicating a left or right response. During stop trials, a tone was played at some delay (SSD) and instructed participants to suppress the indicated response. The presented face stimulus contained allSF, only LSF, or only HSF information. Prints underestimate the contrasts used in the current experiment, especially for the LSF and HSF pictures. The letters displayed on each face are only included here for clarity and were not on top of the faces during the experiment.

While a growing body of literature has focused on the role of sensory evidence during action planning and decision-making, effects on action withdrawal or response inhibition remain largely unknown. Previous work in the field of action control has shown that both the process of response initiation and inhibition can be affected by a common factor such as the introduction of response conflict [48], selective attention [47], changes in motivation [29], [49], and emotions [50], [51]. However, to the best of our knowledge, no previous study has demonstrated how the quality of visual sensory information affects voluntary controlled processes such as response inhibition, and initiation. In this first experiment, we assessed whether the type of spatial frequency information presented affects the efficiency of action control during response inhibition and initiation. To this end we used a modified stop-signal paradigm where traditional go stimuli (i.e., arrows) were replaced by images of male or female faces, containing either allSF, only LSF, or HSF information (Figure 3).

Performance in the stop-signal task is often interpreted through the horse-race model, which asserts that initiation and inhibition processes are independent and compete for the first finishing time [31], [34], [35]. That is, if the inhibition process finishes before the initiation process a planned response is inhibited. However, if the initiation process finishes first, the planned response escapes inhibition and a response is produced [52]. The time needed to inhibit a planned response is referred to as the stop signal reaction time (SSRT) and can be estimated from the proportion of correct stop trials (i.e., successful inhibition) and the distribution of reaction times (RT). For the current study, we predicted the removal of spatial frequency information to slow the ease of information accumulation and therefore affect decision processes that underly gender indentification in a stop-task. The greater uncertainty that comes with the slower accumulation should lead to (1) a longer finishing time for the go processes, and (2) a shorter finishing time for the inhibition process (lower SSRT), due to the increased motivation to stop in an uncertain situation. We predict the strongest effect on both go and stop processes, when only detailed information (HSF pictures; processed at a very low rate in the visual system) is presented.

#### Effects of spatial frequency information on response inhibition

**Figure 4:**
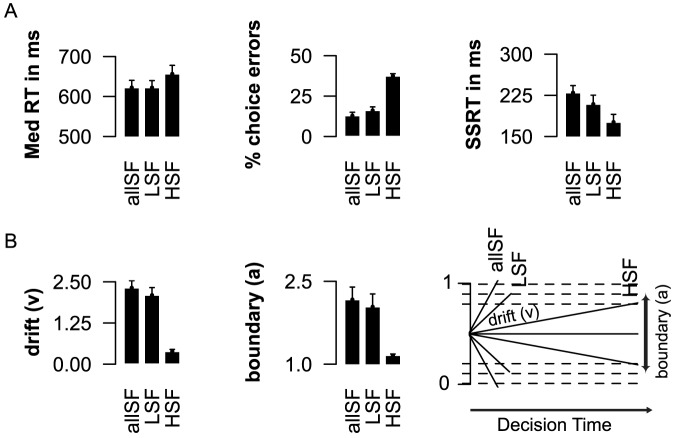
Experiment 1: Effects of spatial frequency information during the visual stop task. A) Both Go RT (left panel) and 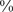 choice errors (middle panel) increased when spatial frequency information was degraded. There right panel shows how the efficiency to withdraw a response (SSRT) improves, when categorization is more difficult. B) HDDM individual subject parameter estimates. When spatial frequency information was removed, the rate of information accumulation, “drift rate” (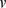) decreased (left panel), whereas the decision “boundary” (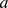) to categorize the face stimuli on time was lowered (middle panel). The right panel illustrates how lower drift rates, and decision boundaries together can result in prolonged RT and more errors.

Table 1 shows the behavioral data for experiment 1. During go trials, reaction times were increased in the HSF condition (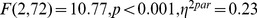; Figure 4A left panel), while the percentage errors decreased over the three conditions from allSF to HSF (
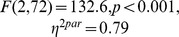
; Figure 4A middle panel). Notably, model selection indicated that a HDDM where the rate of information accumulation, “drift rate” (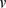), response “boundary” (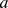), and non-decision time (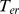) were all allowed to vary across the spatial frequency conditions, best explained the observed RT differences (Table S1 and Figure S1). In line with our predictions, inspection of the “drift rate” parameters (Table 2), indicated that the rate of information accumulation (i.e., how fast information is gathered about the presented stimulus) decreased across conditions with the strongest effect for HSF pictures (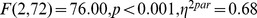; Figure 4B left panel). Interestingly, further inspection of individual subject HDDM parameters indicated a decease of response “boundary” (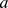) when information accumulation is slowed (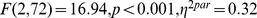; Figure 4B middle panel), with an additional increase in non-decision times (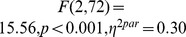). Therefore, the removal of spatial frequencies not only affects the decision process (i.e., 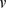 and 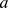 parameter), but also the very early encoding of stimulus information or muscle initiation plans (non-decision time; 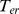).

**Table 1:** Behavioral overview of all visual stop task experiments.

	Go RT	% Choice error	Failed Stop	SSRT	SSD	P(resp)
**Exp.1**						
allSF	622.4±109.3	12.9±12.8	591.1±94.4	229.8±78.7	417.2±152.1	0.56±0.07
LSF	622.3±106.3	16.1±13.1	602.2±108.7	209.1±98.7	428.8±168.9	0.54±0.06
HSF	656.6±129.2	37.5±8.7	647.1±134.1	176.2±85.9	495.4±155.2	0.55±0.07
**Exp. 2**						
*no cue*						
allSF	595.1±93.4	9.4±5.7	581.9±76.0	283.7±118.8	330.9±141.8	0.56±0.08
LSF	604.9±91.2	14.3±8.4	587.7±96.5	239.3±111.7	381.0±137.1	0.56±0.09
HSF	646.5±101.5	37.9±9.4	638.1±86.8	220.0±112.4	449.9±160.8	0.58±0.06
*with cue*						
allSF	573.8±91.9	10.0±6.1	564.9±56.8	270.5±77.4	323.4±133.5	0.57±0.06
LSF	581.2±82.4	13.9±7.5	578.3±78.7	241.0±101.1	361.3±127.8	0.56±0.07
HSF	616.6±79.4	36.5±9.1	629.6±94.8	229.4±111.1	413.3±151.3	0.58±0.06

Values are mean 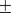 SDs. The cue refers to the spatial frequency information that was (or not) provided prior to the stimulus. SSD = stop-signal delay in milliseconds (ms); SSRT = stop signal reaction time in ms; Go RT = median reaction time in ms; Failed Stop = RT in ms for failed stop trials; P(resp) = probability of responding given that a stop signal was sounded.

**Table 2:** Estimated HDDM parameters for all visual stop-task experiments

	Subject	Estimates		Group	Estimates	
	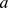	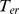	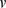	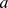	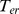	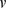
**Exp.1**						
allSF	2.17±1.37	0.29±0.07	2.32±1.31	1.83±0.18	0.28±0.02	2.32±0.28
LSF	2.04±1.41	0.30±0.08	2.10±1.39	1.70±0.11	0.29±0.02	2.09±0.27
HSF	1.16±0.14	0.36±0.08	0.39±0.37	1.16±0.17	0.35±0.02	0.39±0.28
**Exp. 2**						
*no cue*						
allSF	1.57±0.63	0.34±0.05	2.33±0.62	1.45±0.17	0.33±0.02	2.33±0.36
LSF	1.28±0.31	0.37±0.05	1.84±0.62	1.24±0.09	0.36±0.01	1.84±0.31
HSF	1.08±0.15	0.39±0.05	0.45±0.43	1.07±0.07	0.39±0.01	0.45±0.30
*with cue*						
allSF	1.33±0.38	0.35±0.04	2.25±0.76	1.28±0.08	0.35±0.01	2.25±0.30
LSF	1.22±0.25	0.37±0.04	1.88±0.66	1.19±0.07	0.37±0.01	1.88±0.29
HSF	1.07±0.12	0.38±0.05	0.50±0.41	1.06±0.05	0.38±0.01	0.50±0.30

Values are mean 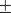 SDs for all parameters of the most representative drift diffusion model during each visual stop task. Note that for group estimates, SD is computed from the posterior distribution of parameter estimates.

Central to our hypothesis, we next examined how the type of visual frequency information available affects the efficiency of response inhibition (Figure 4A right panel). Importantly, stopping performance (SSRT) improved (decreased) when the categorization of stimuli became more difficult (
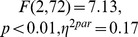
). Furthermore, the type of frequency information (from allSF to HSF) increased average SSD (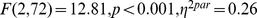), and RT on failed stop trials (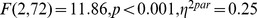). No effects of spatial frequency information were found on P(resp|stop).

Finally, supporting the independency assumptions of the horse race model [34], [35], we found no correlation between the degree of slowing during go trials and decreasing SSRTs (all 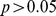). Note that, while this relationship suggests that effects of spatial frequency information on RT and SSRT are functionally independent, it does not automatically map onto assumptions of stochastic independence (which asserts that RT and SSRT within a given trial are independent, and which is crucial to the logic associated with the race model). Nonetheless, when pooled across trials and correlated across individuals, the lack of functional dependence does at least suggest that the information contained by the different spatial frequencies differentially affects the speed of processing of go and stop responses.

Experiment 1 examined how the spatial frequency of available information affects the efficiency of action control. Consistent with the literature [20], [53], [54], our results indicated that categorization of faces becomes increasingly difficult as the spatial frequency of available information ranges from the full spectrum (allSF) to only LSF, and HSF. Across the frequency conditions, the speed of information accumulation decreased; while both the time to respond (go RT) and the percentage of choice errors increased. The increased number of errors was further reflected in lowered response boundaries when only HFS information was presented. That is, when stimulus information was processed slowly, participants decreased their response “boundaries”, and required less information to reach a response decision.

Importantly, the type of spatial frequency presented also affected the efficiency to withdraw a response. That is, stopping performance was more efficient (faster) when stimuli were more difficult to categorize. We will discuss this point further in the general discussion. Together, these results suggest that the type of spatial frequency available affects both response inhibition and initiation. Moreover, the lack of a relationship between the degree of slowing and improvements in the efficiency of stopping, suggested a differential role for available spatial frequency information during stop and go processes.

### Experiment 2

**Figure 5:**
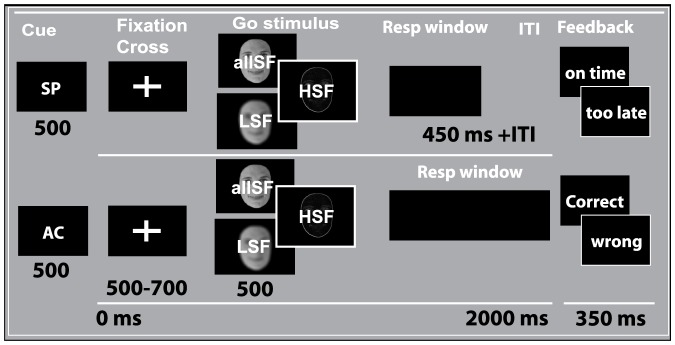
Visual speed-accuracy task. Prior to each trial a cue was presented indicating a speeded (“SP”) or accurate (“AC”). The presented face stimulus (male or female) indicated a right- or left- response, and contained allSF, only LSF, or only HSF information. After a speed cue, trials were followed by feedback stating “on time” if responses were below 450 ms, and “too late” if responses were larger than 450 ms. Accuracy trials were followed by feedback stating either “correct” if participants responded correct or “incorrect” when an error was made. For simplicity, the cue and feedback text are displayed here in English, in the real experiment these were provided in Dutch. Prints underestimate contrasts used in the actual experiment, especially for the LSF and HSF pictures. The letters displayed on each face are only included here for clarity and were not on top of the faces during the experiment.

The goal of our second experiment was threefold. First, we examined the reliability of effects found in experiment 1. Second, we examined whether effects obtained in the stop-task could be generalized to the field of strategic decision-making. Although the role of sensory evidence has been studied in numerous decision-making studies [9], [55]–[57] stimuli with a clear distinction in processing speed, as obtained by manipulating spatial frequencies, have thus far not been used to examine underlying latent processes that affect strategic decision making. In the stop task, participants either need to produce a response (go task) or withdraw a response (stop-task). However, the stop-task in experiment 1 cannot address how spatial frequency information affects strategic behavior (fast or accurate response strategy) within one category (e.g., response initiation). We therefore examined how the type of available spatial frequency information would affect response (initiation) strategies when instructed to respond with a deadline (Figure 5). Finally, it is known from attention and neuroimaging literature that when given prior knowledge, participants are capable of focusing on specific features that are differential to the visual system [58]–[62]. Therefore, an additional interesting question is whether advance knowledge about the type of information available affects controlled performances. We addressed this question in a more explorative analysis, by asking how prior knowledge about the type of information to be received (allSF, LSF, HSF) has an impact on decision-making and action control. In total, participants performed a set of four experiments comprising: two visual-stop tasks, and two visual-speed accuracy experiments (with or without prior knowledge about information type).

#### How advance knowledge of spatial frequency type affects stop signal task performance

**Figure 6:**
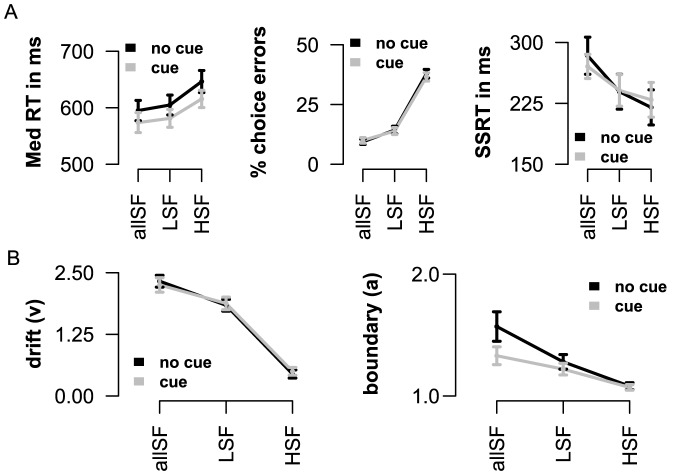
Experiment 2: visual stop task, with or without prior information about spatial frequency type (cue). A) In both experiments Go RT (left panel) and 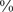 choice errors (middle panel) increased when spatial frequency information was removed. Again, response inhibition was more efficient when categorization became more difficult (right panel). Prior knowledge about frequency type improved only response initiation (Go RT). B) Parameter estimates obtained from HDDM showed a decrease in “drift rate” (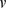) (left panel) and decision “boundaries” (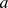) (right panel) with the removal of spatial frequency information.

Figure 6 displays the main effects found in experiment 2, with the cue and non-cue visual stop tasks. Complementing experiment 1, visual frequency information increased both median RT (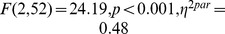) and percentage choice errors (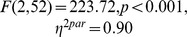). Formal diffusion model analysis again indicated that a model where the rate of information accumulation (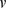), response “boundary” (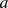), and non-decision time (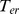) are all allowed to vary across spatial frequency conditions (information), best explains the observed data in both visual stop tasks (Table S1 and Figure S1). Repeated measures ANOVA indicated a decrease of “drift rate” (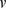) across the three information conditions (
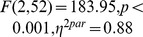
; Figures 6B left panel). Replicating findings from experiment 1, response “boundary” (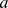) was found to decrease across the frequency conditions (
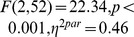
; Figures 6B right panel), while 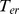 increased (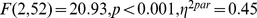). Importantly, again, the efficiency to stop a response improved when categorization of stimuli was more difficult (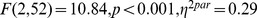). No relationship was found between the rate of response slowing during go trials and improvements in the efficiency of stopping.

Notably, prior information about the type of frequency information (to be received) only affected the speed of response initiation (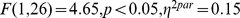; Figures 6A left panel). Inspection of HDDM parameters, between the cue and non-cue experiments, showed an interaction between cue and response “boundary” (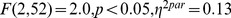), where response “boundary” differences (diff = non cue 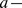 cue 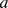) decreased from allSF (diff = 0.24), tot LSF (diff = 0.06), and HSF (diff = 0.01). No further main effects of cue, or interactions with spatial frequency information and cue, were found when inspecting the other behavioral parameters (all 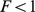). The current results replicate findings from experiment 1, and show that prior visual information generally speeds RT, without reducing any differences in categorization difficulty or stopping efficacy. We will return to this point in the general discussion.

#### Effects of spatial frequency information on speeded or accurate response strategies

**Figure 7:**
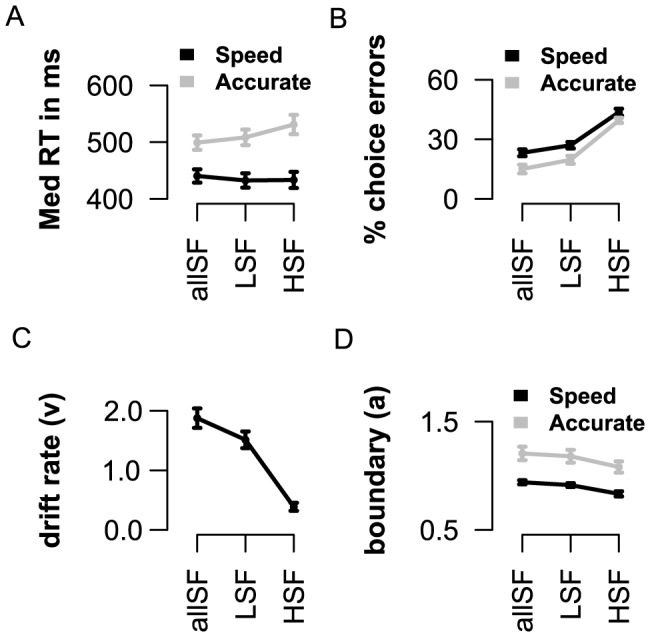
Effects of spatial frequency on decision-making. A) The difference between speeded- and accurate- reaction times (SAT) increased over the three frequency conditions. B) Overall, error rates increased over the three conditions while the SAT in 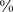 choice errors became smaller between fast and accurate trials. Computational modeling indicated that the speed of information accumulation is decreased over the three conditions (C). The amount of information required to produce a response is higher when instructed to respond accurately, but decreases across the frequency conditions for both speeded and accurate instruction trials (D).

Table 3 gives an overview of the behavioral data in both speed-accuracy tasks. Inspection of response times indicated a significant interaction between context (speed, accurate) and information (allSF, LSF, HSF) on median RT (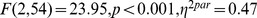; Figure 7A), and percentage choice errors (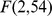 = 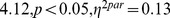; Figure 7B). Analysis of within-subject contrasts indicated that the difference between speeded and accurate responses increases over the three information conditions for correct RT (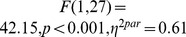), while decreasing for percentage choice errors (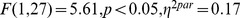). A HDDM model where response “boundary” (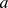) was allowed to vary across context (speed, accurate) and spatial frequency (allSF, LSF, HSF), while “drift rate” (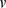) and non-decision time (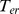) were only allowed to vary across the frequency conditions, best captured strategic response adjustments in both speed-accuracy tasks (Table S2 and Figure S2). Consistent with the observations in the visual stop tasks, the rate of information accumulation (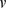) decreased across the three frequency conditions (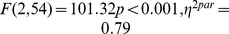; Figure 7C), while 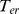 increased for the HSF pictures (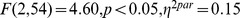; Table 4). As shown in Figure 7D, response “boundary” (i.e., how much information does one require before producing a response) was higher for accurate responses (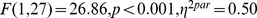), when compared to the speeded condition. Both for speeded and accurate instruction trials, response boundaries were lowered when only HSF information was presented, and gender categorisation was most difficult (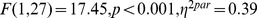). We obtained no effects of prior knowledge (about frequency) on speeded or accurate responses (all *F_s_*<1).

**Table 3:** Behavioral overview speed-accuracy experiments.

		Exp.2:	mixed-design		Exp.3:	blocked-design		
	*no cue*		*with cue*		*no cue*		*with cue*	
	Speed	Accurate	Speed	Accurate	Speed	Accurate	Speed	Accurate
**allSF**								
Median RT	440.4±62.0	499.2±67.4	447.0±62.0	496.5±70.8	392.1±73.7	492.9±68.7	396.6±65.8	487.0±63.1
% Choice errors	23.2±12.1	15.0±9.2	23.8±11.5	16.2±8.6	27.9±11.1	16.0±9.3	28.8±11.9	14.5±8.3
**LSF**								
Median RT	432.5±66.6	508.4±72.7	437.5±70.8	504.4±76.3	401.2±76.1	505.4±72.4	403.3±72.9	505.3±67.5
% Choice errors	27.0±11.1	19.7±8.5	25.8±10.6	18.8±8.9	33.0±10.3	19.8±8.6	32.7±8.5	20.3±8.4
**HSF**								
Median RT	433.4±75.4	531.1±90.3	434.8±74.6	527.4±88.9	406.0±77.9	524.9±72.7	415.0±68.7	534.7±91.7
% Choice errors	43.7±7.5	39.6±9.3	43.7±6.9	40.1±9.5	44.4±6.1	37.7±7.2	43.5±6.4	36.7±7.2

Values are median RT given in ms, and errors in percentages (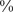) 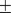 SDs.Cue refers to the spatial frequency information that was (or was not) provided prior to the stimulus.

**Table 4:** Estimated HDDM parameters during decision-making.

	Subject	Estimates		Group	Estimates			
	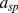	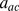	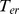	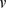	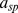	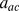	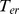	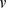
*no cue*								
allSF	0.94±0.10	1.21±0.33	0.27±0.08	1.88±0.86	0.93±0.05	1.17±0.06	0.26±0.02	1.87±0.23
LSF	0.91±0.09	1.18±0.31	0.26±0.09	1.51±0.74	0.91±0.04	1.14±0.06	0.24±0.02	1.51±0.22
HSF	0.83±0.13	1.08±0.27	0.29±0.10	0.39±0.36	0.82±0.04	1.05±0.05	0.26±0.02	0.39±0.22
*with cue*								
allSF	0.92±0.09	1.14±0.30	0.28±0.07	1.81±0.79	0.91±0.05	1.11±0.06	0.28±0.03	1.81±0.20
LSF	0.90±0.10	1.15±0.30	0.27±0.09	1.64±0.75	0.89±0.05	1.11±0.06	0.25±0.02	1.63±0.20
HSF	0.83±0.12	1.06±0.26	0.29±0.08	0.36±0.34	0.82±0.04	1.03±0.05	0.27±0.03	0.37±0.20

Mean 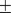 SDs for all estimated parameters. 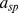 = response boundary (“level of cautiousness”) for speed trials; 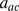 = response boundary for accuracy trials. Note that for group estimates SD is computed from the posterior distribution of parameter estimates.

Findings from experiment 2 replicate and extend findings from experiment 1 by suggesting that the type of available visual frequency information differentially affects the regulation of response inhibition and decision-making.

During the visual inhibition task, lower levels of available visual information were again related to longer reaction times, increased error rates, and most notably shorter inhibition times. In addition, we found that the removal of spatial frequency information slows the rate of information accumulation, and subsequently decreases the criterium for the amount of information needed to produce a response on time. Importantly, prior information about the type of stimuli to be received speeded correct responses without affecting the efficiency to stop. This latter finding further supports theoretical accounts of the horse race model, and suggests that the effects of sensory information on SSRT are independent from the level of response preparation. Additionally, this effect suggests that participants are capable of using the spatial scale of information as an attention feature by priming the high and low spatial frequency systems selectively [60], [63], [64]. Possibly, this information can then be used to speed up response initiation when stop-signals are omitted and response initiation becomes relevant.

When participants were engaged in a visual decision making task, the type of available information interacted with the speed-accuracy tradeoff (SAT) for correct and incorrect responses. Interestingly, formal reaction time analysis, using HDDM, indicated that the rate of information accumulation and non-decision time are modulated over the three frequency conditions, while response “boundary” is altered between speeded and accurate instruction trials, *and* across spatial frequency conditions. Taken together, these findings imply that when perceptual information is processed at a slow rate participants are more ready to withhold their responses, and lower their criteria to reach a decision while maintaining a differential strategy toward speeded or accurate responses. Notably, no effect of cue was found in the visual decision making task.

### Experiment 3

In experiment 2, prior information about the type of spatial frequency information speeded up go responses in the visual inhibition task, but not in the visual decision-making task. One possible explanation for this discrepancy might be the overall longer RT during the inhibition task. The faster response times in the decision-making task (even during accurate trials) might be a direct result of the mixed design used in experiment 2, where accurate and speeded trials are randomly intermixed. An alternative explanation for the discrepancy might be that participants ignore the information cue, because the context cue is deemed more relevant on a trial-to-trial basis. Therefore, a blocked design where differences between speeded and accurate trials become even more apparent, while the information cue becomes more relevant on a trial-to-trial basis, might reveal comparable effects of prior information on response initiation in the stop and speed-accuracy task. Experiment 3 examined whether prior knowledge about the type of spatial frequency information affects decision-making in a blocked design, containing mini blocks of either speed or accurate instruction trials.

#### Advance knowledge of spatial frequency type during speeded or accurate responses

In line with previous findings, the speed-accuracy tradeoff increased over the three information conditions for correct responses (
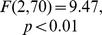
, 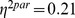), while the difference in error rates decreased (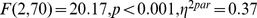). Again, prior information about the type of visual frequency had no effect on the speed of correct or erroneous responses (all 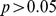).

Experiment 3 examined the potential effect of prior knowledge on correct and incorrect responses in a blocked speed-accuracy design. In line with the initial observations of experiment 2, prior knowledge about the type of spatial frequency information had no effect on response strategies during fast or accurate responses. Therefore, prior information might only be relevant when it motivates one task set over the other. That is, during the stop task participants have to prepare a go response, but also be motivated to withhold this response in time if a stop signal is sounded. Prior information about the type of information is then relevant for the go task, but not the stop task. In the speed-accuracy task prior knowledge is relevant for both speeded and accurate responses. We will discuss this point in more detail below in the general discussion.

## Discussion

The present study examined how bottom-up sensory processing of spatial frequency information interacts with strategically controlled behavior during inhibitory control and decision-making. We found that the available type of spatial frequency information, differentially, but robustly, affects the efficiency to suppress a planned response as well as the balance to react quickly or accurately.

During all visual stop-tasks, formal modeling of the reaction time data indicated that the quality of sensory information, extracted from the stimulus, decreased with degraded levels of spatial frequency information; consequently resulting in prolonged reactions times, decreased accuracy, and lowered response boundaries. Importantly, degraded types of frequency information were also related to more efficient inhibition times. The lack of a relationship between effects found on go (degree of slowing) and stop (improved stopping; SSRTs) trials indicated that degraded frequency information not only prolongs response planning, but independently also speeds response withdrawal. This differential effect on go and stop trials goes well with the dominant horse-race model [34], [35], [65], which asserts that the stop and go process are independent, and compete for the first finishing time [52]. Potentially, the greater certainty that comes with the accumulation of fast low spatial frequency information shortens the finishing time of the go response, while the cost of suppressing a correct response is increased [66]. That is, when visual information has endowed us with a fast and secure response plan, we are more slow and reluctant to withdraw the investment to abort and suppress the prepared response. On a more general level, these results suggest that when visual information is more complete, and swiftly processed, response selection processes gain both in time and accuracy, while the readiness to stop a correct response is reduced and therefore takes longer to process [29], [49].

A second goal of this study was to examine how spatial frequency information would affect other types of controlled behavior such as strategic decision-making. While the stop-experiments focused on two separate response categories (i.e., stop and go), we used decision-making paradigms to examine how strategic responses *within* one category (i.e., speeded and accurate go), are affected by the type of visual frequency information presented. Again, modeling of response times suggested that the rate of information accumulation and the amount of information needed to reach a decision, both decrease over the frequency conditions during all trials. In addition, participants were more cautious and required more information when motivated to respond accurately (as compared to fast instruction trials). Reaction time analysis indicated that when categorization was correct, response time differences between speeded and accurate trials (speed-accuracy tradeoff; SAT) increased with the decay in stimulus strength. Notably, an opposite effect was found when categorization was incorrect, as SAT differences in percentage choice errors became smaller with reduced stimulus quality. Thus, while response boundaries were increased as a function of motivation (respond accurately), the parallel decrease of response boundaries across frequency conditions prevented a sufficient improvement in accuracy rates when the pace of information processing was very low (i.e., during HSF trials).

An important observation that stands out in all experiments is the robust effect of frequency type on the speed of information processing and response “boundaries”. That is, in all experiments, the rate of information processing consistently decreased across the three frequency conditions (allSF, LSF, HSF; with the strongest decrease from allSF to HSF), while the criteria for information accumulation was lowered. Previous work has demonstrated differential effects of high and low spatial frequency filters on simple choice RT [20], emotional processing of faces [54], and face perception [21], [22], [67]. However, while HSF and LSF stimuli have been found to produce differential effects on performances, the direction of these effects has not always been conclusive. Therefore, theories have proposed that while gender judgments might rely equally on LSF and HSF information, specific demands of the task determine the importance and use of each frequency band [23], [68]. In the current study we observed stronger effects when presenting only HSF information (as compared to LSF information) on reaction times, error rates, stopping performances and strategic decision-making. One explanation relates this effect to the speed of information convention in the brain, by suggesting that different neural pathways in the visual system are dominated by to different ranges of spatial frequencies [24], [25]. Specifically, detailed HSF information is thought to travel through the ventral cortical visual stream via the slow parvocellular channels, while the fast and more primitive magnocellular pathways carry LSF information. Indeed, formal analysis of the reaction time data supported this theory and indicated that HSF information is processed at a slower rate than LSF information. Therefore, our findings, in conjunction with the published literature, demonstrate a robust effect of spatial frequency information on performance and further underline the importance of speed in sensory processing during voluntary action control.

The final goal of the current experiment was to explore how prior knowledge about the type of future frequency information impacts on motivational or inhibitory performance. In the stop-signal task, prior information about frequency type advanced response production, but it had no effect on stopping, “drift rate”, response “boundary”, errors, or performance differences between frequency categories. By contrast, no effects of prior information on response production were found on speeded or accurate responses in the visual decision making tasks. One possible, but suggestive, explanation for this dissociation between effects on response production might be the relevance of the cue for the response that is prepared [60], [69]. In the stop-task, two separate response sets are prepared (go and stop); precue information about the type of visual information to be received is directly relevant *only* for go responses, *not* for response inhibition. Therefore, targeted top-down attention to specific sensory features (e.g., through the parvo- or magnocellular pathways) could advance the finishing time of response production, without affecting the efficacy to stop. By contrast, the relevance of the cue for strategic decision-making is less clear and advantageous, because both speeded and accurate responses fall within one and the same response category (i.e., response production) and cannot be independent.

Alternatively, the use of cue information might be related to the speed of response production. Due to the possible need of stopping, reaction times for response initiation were slow and averaged around 600 ms in the stop-signal tasks. Potentially, cue information might be especially beneficial to advance the finishing time of the go process when response initiation is built up slowly. Consistent with this theory, overall reaction times in the speed-accuracy tasks were much faster and averaged around 400 ms for speeded trials, and 500 ms for accurate trials. When participants are given a speed instruction, the buildup of response initiation might already be too fast to profit from the information cue. Accordingly, when instructions emphasize accuracy over speed, cue information that leads to faster responding might be fully ignored. Critically, however, more work on the role of sensory expectations in action control and decision-making is required for a full comprehension of current cue results. A crucial question for further research in this field is how or whether prior knowledge about sensory information interacts with top-down attention during visual decision-making and response inhibition [60], [63], [70].

In conclusion, the use of manipulated spatial frequency information in two prominent response control paradigms indicated a strong interplay between bottom-up sensory processing and strategic behavior. Importantly, detailed information that was processed at a slow rate had the strongest effects on all strategic actions. In more general terms, the current results suggest that the quality of visual information has a differential effect on the deliberate production and inhibition of planned actions.

## Methods

### Stimuli

For all experiments a total of 30 grayscale full-front pictures of unfamiliar faces posing with a neutral expression (half male, half female), were selected from the Radboud Face Database [71]. Faces had neither hair nor glasses and were trimmed to remove all external features (neck, hairline). Three versions of each picture were then made (Figure 2), to manipulate the type of spatial frequency information available in the pictures for sex categorization (male, female). In all LSF pictures, high spatial frequencies were removed by convolving the image with a symmetric Gaussian low-pass filter with a size of 20 pixels. The high spatial frequencies (e.g. the edges) were isolated by applying a range-filter that returned the difference between the maximum and minimum values (using dilation and erosion function), in a 3×3 pixel neighborhood. The filter is therefore capable of removing cycles of slower than 0.13 degrees of visual space. This resulted in a total of 90 pictures where each picture had one version containing: 1) all information (allSF), 2) only global information (low spatial frequency information, LSF) or only the local edge information (high spatial frequency information, HSF). Note that, the face picture displayed in the figures is an example face, and not one that was actually used during the experiments. The person displayed in these figures has given written informed consent, as outlined in the PLOS consent form, to publication of her photograph.

### Experiment 1

#### Participants

Thirty-eight adults (8 male; mean age 22 years, range 18–9 years) participated in this study. In accordance with the Declaration of Helsinki, all participants provided written consent prior to the experiment. The ethics committee of the University of Amsterdam approved the experiment and all procedures compiled with relevant laws and institutional guidelines. All participants had normal or corrected-to-normal vision.

#### Task and Procedure

On each trial, a white fixation cross was displayed on a dark computer screen followed by a male or female face stimulus (Figure 3), indicating a left- or right-hand index finger key-press (i.e., the A or L button on the keyboard). In order to manipulate the available frequency information we presented faces with all (allSF), only the low (LSF) or only the high (HSF) spatial frequencies. All faces were presented at 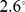 from the centre. Each face stimulus was presented for a period of 500 ms, and the time between the fixation-cross and the face stimulus ranged from 500 to 700 ms (steps of 50 ms). On 25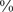 of the trials, the go stimulus was followed by a high tone (stop signal). The stop signal delay (SSD) between the go stimulus and the stop signal was adjusted separately for each stimulus category (allSF, LSF, HSF) according to standard staircase methods to ensure convergence to P(inhibit) of 0.5. Initial SSD was set to 250 ms for all conditions. Instructions emphasized that participants should do their best to respond as quickly as possible while also doing their best to stop the response when an auditory stop signal occurred. Each trial had a fixed duration of 2000 ms. If participants had not responded within a time window of 1000 ms after go stimulus presentation; feedback stating “te langzaam” (“too slow”, in Dutch) was presented for 500 ms.

Prior to the actual experiment all participants performed a practice block of 24 trials to familiarize them with the visual stop task. Participants subsequently performed a total of 480 trials (go: 120 allSF 120 LSF, 120 HSF, stop: 40 allSF, 40 LSF, 40 HSF) in two blocks of each 240 trials. The order of the mapping rules for male and female faces varied across participants, and was reversed across the two blocks.

#### Hierarchical Bayesian Drift Diffusion model

Based on go trial RT distributions of both correct responses and errors, the formal Ratcliff drift diffusion model (DDM) can disentangle the speed of evidence accumulation, “drift rate” (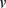), the variability of evidence accumulation (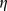), the amount of evidence needed for a decision (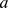), the starting point of evidence accumulation (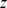), the variability of this starting point (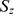). Together these parameters generate a distribution of decision times (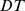). However, observed reaction times (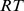) are also thought to contain non-stimulus specific components such as response preparation and motor execution, which combine in the parameters non-decision time (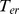), and non-decision time variability (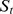). In general, DDM assumes that (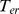) simply shifts the distribution of 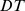 such that: 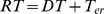 [40], [72], [73]. To analyze the go RT data with the drift diffusion model we used a recently developed hierarchical Bayesian estimation of DDM parameters (HDDM), which allows the simultaneous estimation of subject and group parameters and thus requires less data per subject [39]. To gain a deeper insight into how spatial frequency information affects choice RT (male or female), eight different models were investigated where three DDM parameters of interest were either fixed or varied across the three visual conditions: “drift rate” (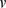), “boundary” separation or response “boundary” (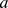), and non-decision time (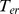). For each model, HDDM obtains a sequence of samples (i.e., a Markov chain Monte Carlo; MCMC) from the posterior of each parameter. Before evaluating the parameters of interest, one must first confirm that the generated samples come from a stationary distribution, and therefore are unaffected by the initial starting value. With complex models (such as DDM) it can take some time before the chain converges from its starting value to a stationary distribution, which can subsequently be used for parameter evaluations (for examples of non stationary chains please see [39]). In the current manuscript we generated 100,000 samples from the posteriors. To make sure that we use only those samples that come from a stationary distribution (and thus are unaffected by starting point) it is common to discard the first samples as “burn in”, and diagnose convergence by running multiple chains. Here, the first 50,000 (burn-in) samples were discarded. Of the remaining 50,000 samples every 10th sample was saved, resulting in a trace of 5,000 samples. Proper chain convergence was assessed using Rhat [74]. This method requires multiple chains to be run, and compares the within chain-variance with the between-chain variance. If all chains converge to the same stationary distribution they should be indistinguishable. For all models, all chains were converged (i.e., all Rhats were close to 1). The best model to describe the data across the three conditions was selected on the basis of Deviance Information Criterion (DIC) [75], reflecting the best trade-off between fit quality and model complexity. For a more detailed description of the HDDM package and underlying equations please see [39].

#### Statistical analysis

The percentage choice errors and median reaction times (RT) were calculated separately for each information condition for go and failed stop trials. Median RT in each condition was calculated after removal of errors. RTs longer than 2.5 above the mean were discarded as outliers (3.34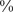 of all go trials). Stop signal reaction time (SSRT) was estimated separately for the allSF, LSF and HSF information condition using the so-called “integration method” [33]. Here, the relative finishing time of the stop-process is modeled as the percentile of the go RT distribution equal to the probability of responding given a stop signal, P(resp|stop). The efficiency of the stopping process, SSRT, is estimated by subtracting the average stop-signal delay (SSD) from the go RT percentile matching P(resp|stop). For example when P(resp|stop) = 0.5, SSD would be subtracted from the median go RT. One way repeated measures Anovas were used to test how the available spatial frequency information (allSF, LSF, HSF) affects performance on go and stop trials. The degree of slowing was defined as the difference between the LSF and All (LSF–All), the HSF and LSF (HSF–LSF), and the HSF and All (HSF–All) condition, for SSRT, RT and 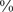 choice errors. One participant was excluded from all analysis, due to a very high average SSD in the HSF condition resulting in negative SSRT estimates, i.e. average SSD was larger than the go RT matching the percentile for P(resp|stop).

### Experiment 2

#### Participants

Thirty healthy adults (10 male; mean age 22 years, range 18–34 years) participated in this study. In accordance with the Declaration of Helsinki, all participants provided written consent prior to the experiment. The ethics committee of the University of Amsterdam approved the experiment and all procedures compiled with relevant laws and institutional guidelines. All participants had normal or corrected-to-normal vision.

#### Task and Procedure

In experiment 2, each participant performed a set of four tasks. The order of task presentation was varied across participants. All stimuli were presented at 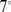 of the screen centre.

The visual stop task followed the same procedure described for experiment 1 (Figure 3), with the exception that stimuli were presented at 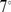 off centre, and responses were now made with the right hand index finger for “left” and right hand middle finger for “right”. The visual cue stop-task was identical to the visual stop task with the exception that now a cue, containing information about the stimulus type, was presented before each trial for 500 ms. Specifically, the cue indicated whether participants would receive a face with all frequency information (“heel”, Dutch for all), only low spatial frequency information (“globaal”, Dutch for global), or only high spatial frequency information (“lokaal” Dutch for local). Note that the cue contained no information about face gender, which was needed for a left- or right- response categorization.

During the visual speed-accuracy task, a cue instructed participants to react as quickly (SN for “snel”, Dutch for fast) or accurately (AC for “accuraat”, Dutch for accurate) as possible to the presented stimulus on a trial-by-trial basis (Figure 5). On each trial, participants decided whether the presented face stimulus (containing either allLSF, LSF or HSF information) belongs to a male (right hand index finger) or female (right hand middle finger). When a speed cue was presented trials were followed by feedback stating “op tijd” (Dutch for on time) whenever response times were below 450 ms, and “te langzaam” (Dutch for too slow) whenever responses exceeded 450 ms. When an accuracy cue was presented trials were followed by feedback stating “goed” (Dutch for correct) when a correct response was given, and “fout” (Dutch for incorrect) when participants gave an erroneous response. The visual cue speed-accuracy task was identical to the visual speed-accuracy task with the exception that now the cue contained information about the stimulus type and the speed-accuracy condition. Specifically, the speed-accuracy cue (i.e., “SN” for fast and “AC” for accurate) was combined with the spatial frequency cue (“heel” for allSF, “globaal” for LSF, and “lokaal” for HSF). For example, on a speed trial where participants would receive an LSF face, the cue would indicate “SN globaal”; and on accuracy trials where an allSF face would be presented the cue would indicate “AC heel”. Again, no information was provided about face gender that was needed for a correct categorization.

In both visual speed-accuracy tasks the cue was presented before each trial, for a period of 500 ms. The order of the presented cues was randomly mixed across trials. Each trial started with a white fixation cross, followed by the face stimulus for a period of 500 ms. The timing between the fixation cross and face stimulus ranged from 500 to 700 ms (with steps of 50 ms). Prior to the actual experiment all participants conducted a practice block of 24 trials to familiarize with the task. Participants subsequently performed two blocks of 240 trials each, with a total of 480 trials, divided equally over the speed (240 total; 80 allSF, 80 LSF, 80 HSF) and accuracy (240; 80 allSF, 80 LSF, 80 HSF) conditions.

#### Hierarchical Bayesian Drift Diffusion model

For both the visual stop- and visual cue stop tasks we defined the same set of eight DDM models with the hierarchical drift diffusion model (HDDM), that were previously used in experiment 1. Recent advances have shown that the slowing of reaction times between accurate and speeded responses (i.e., the speed-acuracy tradeoff “SAT”) is best explained by a shift in the overall amount of evidence needed to make a response [36], [38], [76]. Therefore, to best capture strategic response adjustments between the two instruction conditions (speed and accuracy) we defined a set of sixteen HDDM models (two times the same eight models used in experiment 1). Within these models “boundary” separation (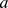) was either fixed or allowed to vary across all three types of stimuli (allSF, LSF, HSF) and both conditions (speed and accuracy); while (consistent with experiment 1) the rate of information accumulation (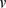), and non-decision time (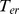) were only fixed or allowed to vary across the three frequency information conditions. As with all stop experiment, we generated 100,000 samples for each model from the posteriors, where the first 50,000 (burn-in) samples were discarded. Of the remaining 50,000 samples every 10th sample was saved, resulting in a trace of 5,000 samples. Proper chain convergence was assessed using Rhat [74]. For all models, all chains were converged (i.e., all Rhats were close to 1). The best model to describe the data across the three conditions was selected on the basis of Deviance Information Criterion (DIC) [75].

#### Statistical analysis

Examination of behavioral parameters followed the same procedures as described in experiment 1, for both visual stop tasks. Outlier rejection of RTs longer than 2.5 SD above the mean, resulted in a data reduction of 3.95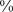 for all go trials in the visual stop task, and 4.84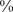 for all go trials in the visual-cue stop task. Repeated Measures Anovas with spatial frequency information (allSF, LSF, HSF) and cue (no cue, with cue) as factors, was conducted to examine how prior information about spatial frequency type affects go and stop performances. We could not obtain reliable SSRT estimates for three subjects, due to very long (average) SSD values during HSF trials. Therefore, these subjects were excluded from all further analysis in the visual-stop task.

Outlier rejection in both visual speed-accuracy tasks resulted in a reduction of 3.47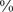 for the task with no information cue, and 2.96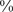 with information cue. Median RT and the percentage choice errors were calculated separately for speeded and accurate trials. Repeated Measures Anovas with information (allSF, LSF, HSF), condition (speed, accurate), and cue (no cue, with cue) as factors, was used to examine how spatial frequency information affects decision processes during motivational response initiation. Two participants were excluded from both speed-accuracy tasks. One participant did not understand the speed-accuracy instructions correctly, leading to very high percentage errors in all conditions (percentage choice errors 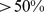) for one block. Another participant made excessive omission errors (omission errors 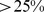) in all conditions.

### Experiment 3

#### Participants

Thirty-nine healthy adults (20 male; mean age 20 years, range 17–30 years) participated in this study. In accordance with the Declaration of Helsinki, all participants provided written consent prior to the experiment. The ethics committee of the University of Amsterdam approved the experiment and all procedures compiled with relevant laws and institutional guidelines. All participants had normal or corrected-to-normal vision.

#### Task and Procedure

Task and procedure for both the cue and normal visual decision-making task were identical to experiment 2 with the following exceptions: 1) Trials were now divided into mini blocks of 30 trials with the same cue for speed or accuracy, 2) participants performed a total of 600 trials, during two large blocks of 300 trials, containing a total of 10 speed (300 trials; 100 allSF, 100 LSF, 100 HSF) and 10 accuracy (300 trials; 100 allSF, 100 LSF, 100 HSF) mini blocks, 3) The practice block contained 40 trials with two mini speed (10 trials) and two mini accuracy blocks, and 4) responses were made with the right- and left- index finger.

#### Statistical analysis

Outlier rejection resulted in a reduction of 3.54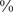 for the non-cue task, and 3.86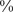 for the cue task. Statistical procedures were identical to the one described for the visual decision-making task in experiment 2. One participant did not complete the task and was excluded from all analysis. Two participants responded at chance level in all conditions and were excluded from all further analysis. One participant made excessive omission errors (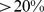) in all conditions, and was also excluded from all further analysis.

## Supporting Information

Figure S1 Examples of posterior predictives (grey lines) from the winning HDDM, on top of the observed normalized reaction time distributions (black) for a representative participant. Errors have been mirrored along the x-axis to display correct and incorrect RT distributions in one plot. A) visual stop task experiment 1, B) visual stop task experiment 2, C) visual cue stop task experiment 2.
(EPS)

Figure S2 Posterior predictives (grey lines) and observed reaction times (black lines) in the visual speed-accuracy task (experiment 2) for a representative participant. A) Accurate instruction trials, B) speeded instruction trials.
(EPS)

Table S1 Model selection with HDDM for the visual stop tasks.
(PDF)

Table S2 Model selection with HDDM during decision-making.
(PDF)
