# Supplementary material for: Spatial Frequency Information Modulates Response Inhibition and Decision-Making Processes
Source: PLoS One. 2013 Oct 21;8(10):e76467. doi: 10.1371/journal.pone.0076467 (PMC3804599; doi:10.1371/journal.pone.0076467)
Supplement: Table S1 — Model selection with HDDM for the visual stop tasks. (PDF) [file pone.0076467.s003.pdf]

Table S1. Model selection with HDDM for the visual stop tasks

**Model**	**Vary**	**Exp. 1**	**Exp. 2: no cue**	**Exp. 2: with cue**
1	a, v, T_{er}	**4447.4**	**337.8**	**−877.6**
2	a, T_{er}	4985.1	716.9	−558.4
3	a, v	5038.7	544.6	−703.8
4	v	5275.6	836.1	−484.0
5	v, T_{er}	6160.7	1504.9	212.5
6	a	6229.7	1554.6	256.9
7	T_{er}	6494.5	1930.8	554.0
8	Fix all	7026.0	1953.8	592.0

Values represent deviance information criterion (DIC). Lower values indicate a better balance between fit and complexity. For clarity values representing the optimal model are printed in bold. [v: drift rate, a: boundary, T_{er}: non-decision time].
