# Supplementary material for: Spatial Frequency Information Modulates Response Inhibition and Decision-Making Processes
Source: PLoS One. 2013 Oct 21;8(10):e76467. doi: 10.1371/journal.pone.0076467 (PMC3804599; doi:10.1371/journal.pone.0076467)
Supplement: Table S2 — Model selection with HDDM during decision-making. (PDF) [file pone.0076467.s004.pdf]

Table S2. Model selection with HDDM during decision-making

**Vary**		**DIC**	
**Information**	**context**	**no cue**	**with cue**
a, v, T_{er}	a	**−1497.0**	**−1978.7**
a, v	a	−983.1	−1568.0
v	a	−925.3	−1455.2
a, T_{er}	a	−591.6	−1052.9
T_{er}	a	−567.3	−991.2
a, v, T_{er}	Fix all	−242.2	−816.3
v, T_{er}	Fix all	−216.3	−700.1
Fix all	a	−11.0	−533.1
a	a	0.4	−550.3
a, v	Fix all	158.9	−466.8
v	Fix all	217.1	−356.9
a, T_{er}	Fix all	574.6	46.7
T_{er}	Fix all	607.0	161.0
a	Fix all	1061.6	484.4
Fix all	Fix all	1069.0	524.9
v, T_{er}	a	1425.4	−1876.5

Information indicates that parameters were allowed to vary across spatial frequency conditions (allSF, LSF, HSF). Context indicates that parameters were allowed to vary across the speed and accuracy condition. For clarity values representing the optimal model are printed in bold. In both tasks, the winning model (with the lowest DIC) allowed drift rate (v), boundary separation (a), and non-decision time to vary across spatial frequency information while boundary separation (a) was additionally allowed to vary across context.
